# Mapping Dysregulation: Prenatal Predictors and Developmental Trajectories of Multiple Regulatory Problems in Early Childhood

**DOI:** 10.1007/s10802-025-01320-2

**Published:** 2025-04-08

**Authors:** Beate Helmikstøl, Vibeke Moe, Lars Smith, Eivor Fredriksen

**Affiliations:** 1 Department of Psychology, Ansgar University College, Kristiansand, Norway; 2 Department of Psychology, University of Oslo, Oslo, Norway

**Keywords:** Dysregulation, Multiple regulatory problems, Cumulative risk, Prenatal risk, Latent change

## Abstract

Regulatory problems, defined as excessive crying, sleeping and feeding problems, as well as pronounced sensory sensitivity, pose a significant source of worry and exhaustion for parents. In this study, we investigate the evolvement of multiple regulatory problems up to 3 years of age. We ask whether a range of maternal prenatal risk factors, including poor mental health, substance use, and sociodemographic risks, predict developmental trajectory in dysregulation from 18 months to 3 years. The sample comprises 748 children and their mothers taking part in the prospective longitudinal community-based study Little in Norway. Utilizing latent change score modelling, we found that prenatal risk factors predicted an increase in dysregulation from 18 months to 3 years (*β* = 0.208, *p* <.001). This association was moderated by child sex, being stronger for boys (*β* = 0.229, *p* <.05) than for girls (*β* = 0.151, *p* <.05). A more fine-grained analysis of subscales showed that sensory sensitivity, eating problems and negative emotionality contributed to these effects, whereas sleeping problems were unrelated (*p* >.05). Results suggest that the association between early risk exposure and child dysregulation may unfold gradually over time. Those born into families with a higher load of prenatal risks are more vulnerable to dysregulation problems extending, or even increasing, into the preschool years.

**Supplementary Information:**

The online version contains supplementary material available at 10.1007/s10802-025-01320-2.

Sleepless nights, periods of excessive crying and feeding problems are well-known concerns for all new parents from time to time. For most families, these problems are transient. For some, the difficulties persist, putting the child at risk for subsequent negative developmental outcomes (Bilgin et al., 2020a, b; Cook et al., 2021; Schmid et al., 2010; Wake et al., 2006). In the research literature such behaviours are often subsumed under the label of *multiple regulatory problems*. This grouping corresponds with the classification of disturbances of sleeping, eating, and crying in the Diagnostic Classification of Mental Health and Development Disorders of Infancy and Early Childhood, DC:0–5 (Zero to Three, 2016). Some argue that sensory sensitivity should be grouped alongside these problems (Carter et al., 2003), and collectively termed dysregulation. The idea is that although these problems look different in their outward expressions, they can be considered jointly as failure to regulate. Dysregulation, or multiple regulatory problems, describe poor modulation that is unusually frequent, intense, or persistent (Hyde et al., 2012), and include issues persisting beyond 4–6 months of age (Wolke, 2019).

Beginning in early infancy, and in co-regulation with their caregivers, infants gradually develop multiple monitoring skills, such as ability to self-soothe, smoothly ingest food, establish predictable sleep-wake cycles, direct attention, and exert sensory-motor control, often referred to as self-regulation (Galling et al., 2023). Regulatory difficulties, such as problems with crying, feeding, and/ or sleeping are normative for infants, with an expected gradual reduction throughout childhood, as regulatory capacity gradually develops. The prevalence of regulatory problems varies across studies, ranging from 3 to 10% (Olsen et al., 2019; Wolke, 2019) up to 20–30% (Hemmi et al., 2011; Schmid et al., 2010; Singh et al., 2021). Higher estimates are common in reports of singular problems. Prevalence decreases when assessing multiple concurrent problems compared to problems in one domain only. Estimates around 7–15% have been reported for two or more such problems simultaneously (Winsper & Wolke, 2014; Wolke et al., 1995).

However, variations in terms of definitions, measurement, time spans, and age ranges across studies raise multiple challenges in reviewing the literature (Reijneveld et al., 2001). Moreover, during the preschool years and beyond, discussions often focus on dysregulated behaviours in a broader context, which typically encompass anxiety, depression, attention issues, and aggression. Although problems with eating, sleeping, and crying are robust predictors of later dysregulated behaviours (Wolke et al., 2023), and by some considered early manifestations of later emerging and more broadly defined dysregulation (Asmussen et al., 2022), variations in definitions and measurement remain a major challenge within this field of research (Hemmi et al., 2011), complicating direct comparison across studies.

Dysregulation posits a source of considerable parental distress, exhaustion and concern. It is one of the most common reasons why parents seek professional help for their infants (St James-Roberts, 2008; Wake et al., 2006; Wolke, 2019), underscoring the need for a better understanding of this condition.

## Developmental Trajectories of Dysregulation in Early Childhood

A few studies have investigated regulatory problems longitudinally to examine how multiple regulatory problems develop over time, and results are somewhat mixed (e.g. Olsen et al., 2019; Sirvinskiene et al., 2012). But in general, less stability is reported when measured from early infancy (Fish et al., 1991; St. James-Roberts et al., 1998; Wake et al., 2006). Regulatory problems seem to show higher stability when assessed beyond the first few months of life (Hemmi et al., 2011). Indeed, in a large-scale study, dysregulation was found to be stable in 38.7% of participants from age 1 to 2 (Briggs-Gowan et al., 2006). Hence, although this still means that most such problems are transient, persistence may be more prominent in late infancy and toddlerhood than in early infancy. Along with these findings, Asmussen et al. (2022) reported that from age 2.5 to 5, 17% of their participants evinced continuing regulatory problems, while an additional 13% exhibited increasing problems. Winsper & Wolke (2014) studied dysregulation at multiple time points from 6 months of age and all the way up to 9.5 years. They reported evidence for stable trajectories, with the most dysregulated infants and toddlers displaying the most dysregulated behaviours in later childhood. Taken together, although regulatory problems in most cases are transient, a subgroup of children seems to struggle with more severe and persisting problems from early on. More studies in this area are warranted, especially studies that may help identify these children at an early stage.

Over time persistently dysregulated infants and toddlers are at elevated risk for a wide array of long-term problems associated with mental health, behavioural adjustment, cognitive and language development, social skills, academic achievements, motor development, socioemotional functioning, and parent-child relationships (Bilgin et al., 2020a, b; DeGangi et al., 2000; Galling et al., 2023; Hemmi et al., 2011; Schmid et al., 2011; Singh et al., 2021; Winsper & Wolke, 2014). Although some point out that long-term associations are not strong (Wake et al., 2006), associations with non-favourable outcomes have been documented even into adulthood (Wolke et al., 2023). This seems to be the case especially for infants with moderate to severe regulatory problems (Cook et al., 2020, 2021; DeGangi et al., 2000; Hyde et al., 2012; Schmid et al., 2010; Wolke et al., 2002), as well as for infants struggling with multiple regulatory problems (i.e. sleeping problems, feeding problems *and* excessive crying) (Hemmi et al., 2011; Winsper & Wolke, 2014; Wolke et al., 2023). Severe and persistent regulatory problems are also associated with more parental stress, parental conflict, and parental psychopathology, as well as relational problems in the mother-child dyad (Papousek & von Hofacker, 1998; Singh et al., 2021; Wake et al., 2006), suggesting disruptions, likely bi-directional, to the whole family system.

### Sex Differences in Dysregulation

The literature on sex differences in child regulation is somewhat mixed. Sex differences in levels and persistence of dysregulation, disfavouring boys, have been reported by some (Lundqvist, 2000; Olsen et al., 2019), while others find girls to be more vulnerable (Braithwaite et al., 2017). Others report no such differences (Briggs-Gowan et al., 2006; Schuetze et al., 2018; Sidor et al., 2013, 2017; Sirvinskiene et al., 2012). It might also depend on the nature of the regulatory problem(s) being examined (Schmid et al., 2011).

However, in studies of prenatal risk exposure on various child outcomes, differential sex effects have been documented (Bale, 2011; Fenger et al., 2024; Gerardin et al., 2011; Hernández-Martínez et al., 2010; Moe & Slinning, 2001; Wakschlag & Hans, 2002)- usually with less favourable outcomes for boys than for girls, although results are not fully consistent (Sutherland & Brunwasser, 2018), and often based on low sample sizes. Sex differences may also be time and context specific (Chaplin & Aldao, 2013; Kim et al., 2015). Some advocate that the prenatal period may represent a larger window of vulnerability for boys, while the prepubertal period may increase vulnerability in girls (Bale & Epperson, 2015). Differences appear to be most prominent in studies of nervous system development and temperament in children (Sutherland & Brunwasser, 2018), which may overlap with regulatory functioning. Recommendations are to include child sex in analyses when studying associations between prenatal stress and child outcomes (Sutherland & Brunwasser, 2018). Furthermore, most of the abovementioned studies pertain to the child`s first year of life. Sex differences in relation to prenatal stressors beyond this age is an underexplored topic.

### Predictors of Dysregulation

Despite efforts to identify predictors of dysregulation, effect sizes are generally small, and the most potent predictor for *later* dysregulation seems to be *early* dysregulation (Fish et al., 1991; Olsen et al., 2019; Schmid et al., 2010; Sirvinskiene et al., 2012). While the underlying aetiology remains unclear, several risk factors have been identified.

Maternal risk factors include psychosocial factors, such as low maternal education, minority status (Olsen et al., 2019), family adversity and psychosocial stress (Schmid et al., 2011), younger maternal age (Sirvinskiene et al., 2012), as well as pre-and postnatal maternal mental health problems (Asmussen et al., 2022; Hyde et al., 2012; Korja et al., 2017; Olsen et al., 2019; Petzoldt et al., 2016; Schmid et al., 2011). In addition, use of alcohol, nicotine, and other substances in pregnancy is frequently reported to elevate the risk of dysregulation (Asmussen et al., 2022; Beauchamp et al., 2020; Eiden et al., 2015; Stroud et al., 2009). First-time mothers, mothers expressing much self-doubt, and those with negative reactions towards pregnancy also tend to report more regulatory problems in their children (Cook et al., 2019; Sirvinskiene et al., 2012). Gestation- related factors specifically relate to preterm birth and/ or low birth weight (Poehlmann et al., 2011; Schmid et al., 2011). There may be a particular vulnerability related to poor foetal growth and immature neurobiological systems, (Figueras et al., 2009; Lammertink et al., 2021) and/ or possibly to preterm infants being more susceptible to negative parenting (Poehlmann et al., 2011). Child risk factors particularly comprise temperamental dispositions, foetal abnormalities, neurodevelopmental vulnerabilities, as well as gene-environment interactions (Bilgin & Wolke, 2016, 2017; Olsen et al., 2019; Poehlmann et al., 2011; Poustka et al., 2015; Schmid et al., 2011; Sidor et al., 2017). Child-parent interactions are typically also described as more stressed and disturbed when the child displays persistent regulatory problems (Papousek & von Hofacker, 1998).

Previous research has been criticized for not sufficiently acknowledging that risk factors tend to co-occur and correlate (Hemmi et al., 2011; Schuetze et al., 2018). Within a transactional framework, dysregulation may be seen as a product of the interplay between various risk factors over time- within the child, in the parents, and in a broader context, wherein both interactional, contextual, intergenerational, and genetic transmissions are plausible (Bridgett et al., 2015). Some have also noted that regulatory problems are associated with an accumulation of organic and psychosocial risks (Papousek & von Hofacker, 1998).

### Accumulation of Risks

The cumulative risk approach rests on the premise that it is the *number* of risks, rather than the *nature* of risks that becomes detrimental (Ettekal et al., 2019). While the presence of a single risk factor may have little or no negative effect on child development, the accumulation of multiple risks over time could have detrimental cascading ripple effects on development, adjustment and mental health- beyond what would be expected by simply adding up the expected effects of each risk factor (Evans et al., 2013; Rutter, 1979).

As risks tend to co-exist, persist, overlap, or even accumulate over time (Evans, 2004; Helmikstøl et al., 2023; Wallander et al., 2019), aggregating risks into a cumulative risk index allows for using one predictor variable instead of multiple correlated predictors, thus avoiding issues of multicollinearity and potential suppression effects (Ettekal et al., 2019; Evans et al., 2013). This enhances stability and predictive power, while also providing a more simplified and parsimonious model that is more easily interpretable. The use of cumulative risk approaches has been subject of much debate, with critics emphasizing the lack of distinction between individual risk factors, and the limited ability of such an approach to identify possible underlying mechanisms, specific pathways or causal effects in associations between early risk and later adversity (Ettekal et al., 2019; Evans et al., 2013). While using individual variables may have the potential to detect specific pathways or uncover underlying mechanisms, a more parsimonious cumulative risk approach is suited to maximize the predictive power, aiding in early identification of families with increased risk for adverse outcomes (Hofstee et al., 2024; McGinnis et al., 2022). Few studies of developmental trajectories of cumulative risk in infancy and toddlerhood have been published, even though this period is often thought of as being the critical and formative years of childhood. We contend that attention should be focused on cumulative risk exposure starting from pregnancy, as this constitutes the initial environment for the baby. Early identification could be of great clinical relevance in preventing problems from escalating. As dysregulation is mostly studied during infancy and toddlerhood, this way of mapping dysregulation represents an approach that is currently underexplored, both in terms of method, theme, and time frame.

### Aims of this Study

We set out to explore whether an accumulation of prenatal risks could predict how dysregulation develops from early toddlerhood (18 months) into the preschool years (3 years), as well as the change in dysregulation between these two time points. The focus on *very* early risk acknowledges a shift of focus, away from specific risk factors affecting the individual, on to seeing the child as part of a complex environment, beginning in pregnancy. This study also tackles some of the criticism raised by previous studies on regulatory problems, such as the lack of prospective studies, and small sample sizes (Schuetze et al., 2018; Wolke, 2019). While most studies on dysregulation focus on the child`s first year of life, studies of cumulative risk have largely focused on school-aged children, applying cross-sectional designs (Evans et al., 2013). The present study, therefore, extends on the existing literature in several ways. First, by exploring prenatal predictors of dysregulation in early childhood. Second, through the exploration of how cumulative risk predicts development over time, rather than merely capturing a snapshot of dysregulation. Thirdly, by investigating this through a prospective design starting in pregnancy. Finally, by exploring this in a period that is less frequently studied in research on dysregulation and cumulative risk. Since dysregulation is often transient, whereas persistent or escalating problems are associated with the most negative outcomes, the possibility of very early prediction of persisting or escalating issues over time offers a valuable and clinically significant contribution to this field.

Specifically, this study aims to (i) investigate to what extent an accumulation of prenatal risks can predict an increase in dysregulation from 18 months to 3 years, and (ii) explore whether an association between prenatal cumulative risk and escalation in dysregulation is moderated by child sex.

## Methods

### Sample and Process

This study uses data from the Little in Norway project, a prospective longitudinal community-based study (Moe et al., 2019). Pregnant women (*N* = 1036) from nine different sites across Norway were recruited during routine follow-up in pregnancy at their local public well-baby clinic. In Norway all pregnant women are offered a minimum of eight free prenatal consultations at such clinics, and participants were recruited to the study by midwives during their first consultation, usually mid-pregnancy. There were no exclusion criteria, but as all questionnaires were administered in either Norwegian or English, mastery of either language was warranted. All women received information on the study prior to participation, and written consent to participation was obtained from all on behalf of themselves as well as for their babies. During pregnancy, 26 pregnant women left the study, and 3 were lost due to stillbirth, leaving 1007 mothers and 1017 babies (including 10 twin pairs) for further follow-up. For a description of the sample, see Table 1.

**Table 1:** Description of the sample at enrolment and the sample included in the latent change analyses at 18 months and 3 years

Characteristic	Mean (SD)/ Proportion Enrollment (*N* = 1036)	Mean/ (SD)/ Proportion 18months/ 3 years (*n* = 748)
Age	29.76 (4.78) range 17–43	29.84 (4.67), range 18–43
Education Middle school High school College/ university (1–3 years) College/ university (4 years +)	3.1% 19.8% 35.7% 41.4%	1.6% 18.3% 38.2% 41.8%
Work status		
Full-time work Part-time work Student Disability/ unemployed/ at home/ other	77.3% 7.4% 11.6% 3.8%	78.9% 6.3% 12.2% 2.7%
Parity First time parent	0.60 (0.78), range 0–5 54.9%	0.57 (0.73), range 0–4 55.2%
Marital status		
Married Living together Single Separated/ divorced/ other Ethnic minority	36.2% 59.7% 2.5% 1.6% 6.1%	35.8% 61.1% 2.1% 0.9% 4.4%

Participants with mother-reported ITSEA data at either 18 months or 3 years were included in the analyses. The number of participants varied from 18 months onwards (*n* = 659) to 3 years (*n* = 453), with a total of 748 children being included in analyses. Dysregulation at 18 months was reported by mothers, while at 3 years both parents were asked to report on this. Correlations between maternal and paternal reports were moderate, (*r* =.51, p = > 0.001) implying that there are variations in perceptions of the child. To avoid confounding change within the child with change due to informant discrepancy, the 93 children with only father reports, were removed from analyses. To evaluate attrition, independent sample t-tests were carried out to compare the mothers who remained in the study at 3 years with those who had dropped out. Mothers who remained in the study were on average slightly older (*M* = 30.42, *SD* = 4.385) than the sample at enrolment (*M* = 29.25, *SD* = 5.010) *t*(1019.40) = -4.017, *p* = < 0.001, and had somewhat lower risk scores on the cumulative risk index (*M* = 1.55, SD1.527 vs. *M* = 1.10, *SD* = 1.250) *t*(1031.169) = 5.139, *p* = < 0.001. In terms of gestational week for the babies at birth, participants at 3 years (*Mweek* = 40.06, *SD* = 1.752) and non-participants at 3 years (*Mweek* = 39.98, *SD* = 1.823) did not differ significantly, *t*(1006) = − 0.677, *p* =.499.

Missing data were handled by applying full information maximum likelihood estimation (FIML).

### Measures

**Cumulative Risk Indicators.** A cumulative risk index (CRI) is not a fixed metric, and the indicators that make up such an index vary across studies, depending on purpose of the study, research design, as well as previously identified risk factors in the research literature. In accordance with existing literature, we included measures on sociodemographics, maternal mental health, and contextual life stress. Although sociodemographic variables, such as minority status or education level, not necessarily constitute risk per se, previous research has identified these as relating to regulatory problems (Olsen et al., 2019). These factors may also co-exist and interact. For instance, ethnic minority status has been associated with elevated risk for mental health issues in the prenatal period in Norway (Shakeel et al., 2015). As our aim was to study prenatal predictors, several pregnancy-specific risk indicators were also added, such as medication/ alcohol/ nicotine/ snus^1^ use in pregnancy, pregnancy-related anxiety, and not wanting to have the baby. The CRI in this study is comprised of 12 risk indicators, all of which were reported, at enrolment in the study, see Table 2. While five of the indicators are dichotomous variables, the continuous variables were dichotomized according to predetermined cut-off scores as recommended in the literature (Ettekal et al., 2019; Evans et al., 2013). The following criteria were applied; Education; high school or less (≤ 12 years) was regarded non-optimal. Depression was assessed by the Edinburgh Postnatal Depression Scale (Cox & Holden, 2003; Murray & Cox, 1990). EPDS ≥ 10 was considered non-optimal, in line with validated clinical cut-off in Norway (Eberhard-Gran et al., 2001). Pregnancy-related anxiety, PRAQ-R (Huizink et al., 2004) was assessed, with a PRAQ-*R* > 30 being regarded as non-optimal. For the PRAQ-R, there is no established cut-off, but a cut-off of 30 leaves roughly 20% of our sample in the risk category. This estimate may be a bit on the conservative side (Chandra & Nanjundaswamy, 2020). Life stress was assessed by Parenting Stress Index, life stress subscale (PSI, LS) (Abidin, 1995). PSI LS ≥ 17 was considered non-optimal, in accordance with the PSI manual for clinical referral (Abidin, 1995, p. 12). Problematic drinking habits were assessed by TWEAK (Russell, 1994; Russell et al., 1996). TWEAK ≥ 2 was regarded as non-optimal, in accordance with empirical findings and common clinical practice (Russell et al., 1996). Adverse Childhood Experiences (ACE) (Felitti et al., 1998) was assessed retrospectively by the ACE scale. ACE > 1 was regarded non-optimal. Although no cut-off exists for the ACE form, studies indicate that exposure to one ACE category significantly increases exposure to additional such categories (Dong et al., 2004). This cut-off has also been applied in previous research (Helmikstøl et al., 2023). All other risk items were framed within a “yes”/ “no” format. Ethnic minority, no intention to co-habit with partner after birth, not wanting this child, daily smoking/ snus use in pregnancy, previous psychopathology, as well as any use of prescribed medication in pregnancy, were regarded non-optimal. These were further dummy coded as 0 (risk absent) and 1 (risk present), in accordance with common cumulative risk practice (Evans et al., 2013).

Distributions of the various prenatal risk factors within our sample are listed in Table 2. The most common risk exposures were adverse childhood experiences, ACE (36.6%); problematic drinking habits, TWEAK (24.5%); low education (22.9%); previous mental health problems (21.7%); and pregnancy-related anxiety, PRAQ-R (19.4%).

**Table 2:** Distribution of dichotomized prenatal risk scores, for the full sample at enrolment and for the sample used in analyses at 18 months/3 years

Risk factor	% risk *N* = 1036	% risk *n* = 748
Low education	22.9%	19.9%
Ethnic minority	6.1%	4.4%
No intention to co-habit after birth	3.7%	2.8%
Pregnancy not wanted	3.2%	3.2%
Daily smoking/ snus use in pregnancy	7.3%	5.5%
Prescribed medication in pregnancy	14%	15.9%
Previous mental health problems	21.7%	21.0%
Pregnancy-related anxiety (PRAQ)	19.4%	17.1%
Depressive symptoms (EPDS)	9.7%	7.1%
Life stress (PSI)	9.6%	10.0%
Problematic drinking habits (TWEAK)	24.5%	24.3%
Adverse childhood experiences (ACE)	36.6%	34.9%

**Dysregulation.***Infant-Toddler Social and Emotional Assessment (ITSEA)* assesses social and emotional functioning in children between 12 and 36 months of age (Carter et al., 2003). Adequate psychometric qualities are documented, even at the lower age limits (Sanner et al., 2016). Parents rate their children on a 3-point scale; “not true”/ “rarely” (0), “somewhat true”/ “sometimes” (1), or “very true”/ “often” (2), with higher scores indicating more regulatory problems. The Dysregulation Domain is composed of subscales on negative emotionality (13 questions), sleeping (5 questions), eating (9 questions), and sensory sensitivity (7 questions), 34 items in total (Carter et al., 2003). ITSEA was first administered at 18 months and again at 3 years. Subscales showed moderate to good internal reliability; negative emotionality α = 0.77 (18 months), α = 0.83 (3 years), sleeping problems α = 0.67 (18 months), α = 0.54 (3 years), eating problems α = 0.72 (18 months), α = 0.78 (3 years), and sensory sensitivity α = 0.50 (18 months), and α = 0.49 (3 years). For the full dysregulation domain, reliability was α = 0.76 at 18 months, and α = 0.83 at 3 years.

**Covariates.** Gestational week and maternal age were added as control variables, and child sex as a moderating variable. Information on gestational week and child sex was obtained from hospital records as reported by health care professionals. Maternal age was based upon self-report at enrolment in the study.

### Statistical Analyses

Preliminary descriptive analyses were run to gain an overview of sample characteristics and distribution of risk indicator scores. Bivariate associations of dysregulation domain and subscales were mapped.

Latent Change Scores (LSC) were modelled to assess developmental change in dysregulation between measurement points. LCS models are considered a subtype of longitudinal structural equation modelling (SEM), and well suited at investigating change and dynamic associations between variables at multiple time points. These models represent a way of investigating how children develop differently. LCS can, though with some limitations, be applied even with only two measurement points (Kievit et al., 2018; McArdle, 2009). Initially we ran an unconditional model estimating change in dysregulation and evaluated model fit. We then added CRI as a covariate to the same model to examine whether our CRI could predict change in dysregulation scores from 18 months to 3 years. This was first performed for the dysregulation domain. As a post hoc analysis, this was repeated for each separate subscale; negative emotionality, eating, sleeping, and sensory sensitivity. To explore whether the association between prenatal risk and change in dysregulation was moderated by child sex, we conducted multigroup comparisons, applying the Satorra-Bentler Scaled Chi-Square Difference Test to account for using MLR as estimator. We used maximum likelihood with robust standard errors to account for non-normal distributions. The model was fully saturated.

All analyses were conducted using Mplus8.3.

### Ethical Considerations

Recruitment and data collection have been approved by the Regional Committees for Medical and Health Research Ethics in Norway (REK, [2011/560]).

## Results

### Descriptive Statistics

Mean level of dysregulation was 0.39 at 18 months and 0.44 at 3 years, with reported mean levels on the various subscales ranging from 0.25 to 0.49, see Table 3. Scores on ITSEA may range from 0 to 2, leaving the mean scores at the lower end, as is to be expected in a community sample. Level of reported dysregulation, including all subscales showed an increase from 18 months to 3 years. Correlations with the CRI are significant at 3 years, but not at 18 months. For a more detailed overview, including associations with maternal age, child sex, and gestational week see Table 3.

**Table 3:**
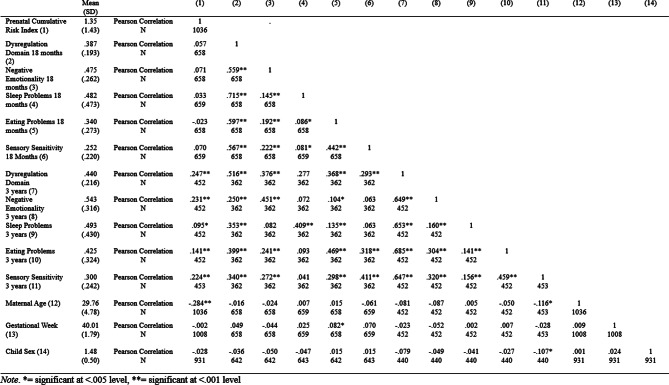
Mean (SD) level and bivariate associations for the prenatal risk index and dysregulation scores at 18 months and 3 years

### Change from 18 Months To 3 Years

To address the first research question of whether CRI could predict an increase in dysregulation from 18 months to 3 years, we applied latent change score modelling (in accordance with Kievit et al., 2018; McArdle, 2009).

First, an unconditional latent change score model was constructed for the full dysregulation domain (*n* = 748) (see supplementary material, Figure S1). There was an increase in dysregulation scores from 18 months to 3 years (intercept of the latent change score factor = 0.205, SE = 0.021, *p* >.001), as well as individual differences in the change scores (variance parameter for the latent change score = 0.033, SE = 0.03, *p* >.001). Further, the rate of increasing dysregulation was larger for those with lower scores at 18 months, as evident from the proportional change regression coefficient (b = -0.396, SE = 0.053, *p* >.001).

We then included CRI as a covariate in the model, with both the initial time point at 18 months as well as the latent change factor being regressed on CRI (*n* = 748). Results showed that higher CRI predicted a larger increase in dysregulation between 18 months and 3 years (β = 0.208, SE = 0.046, *p* =.000). Results indicate a larger increase in dysregulation from toddlerhood and into the preschool years when exposed to an accumulation of prenatal risks. Gestational week, maternal age, and child sex were not significant predictors for this change.

In post hoc analysis, investigating patterns in the subscales constituting the dysregulation domain, latent change scores were then modelled for each separate subscale of the dysregulation domain, identical to the LCA model described above, see Table 4. CRI predicted proportional change in negative emotionality, eating problems, and sensory sensitivity. Associations for sleeping problems were not significant. This implies an increase in regulatory problems over time related to prenatal risk exposure. The model was fully saturated (RMSEA = 0.000, TFI = 1.000, CFI = 1.000, SRMR = 0.000).

**Table 4:** Change in dysregulation predicted by prenatal risks (CRI) from 18 months to 3 years for each subscale of the dysregulation domain of ITSEA

Dysregulation subscale	b	β (S.E.)	*p*
Negative emotionality	0.043	0.189 (0.047)	0.000
Sleeping problems	0.019	0.052 (0.041)	0.207
Eating problems	0.036	0.157 (0.047)	0.001
Sensory sensitivity	0.038	0.207 (0.044)	0.000

### Sex Differences Related To Prenatal Risk Exposure

**Fig. 1:**
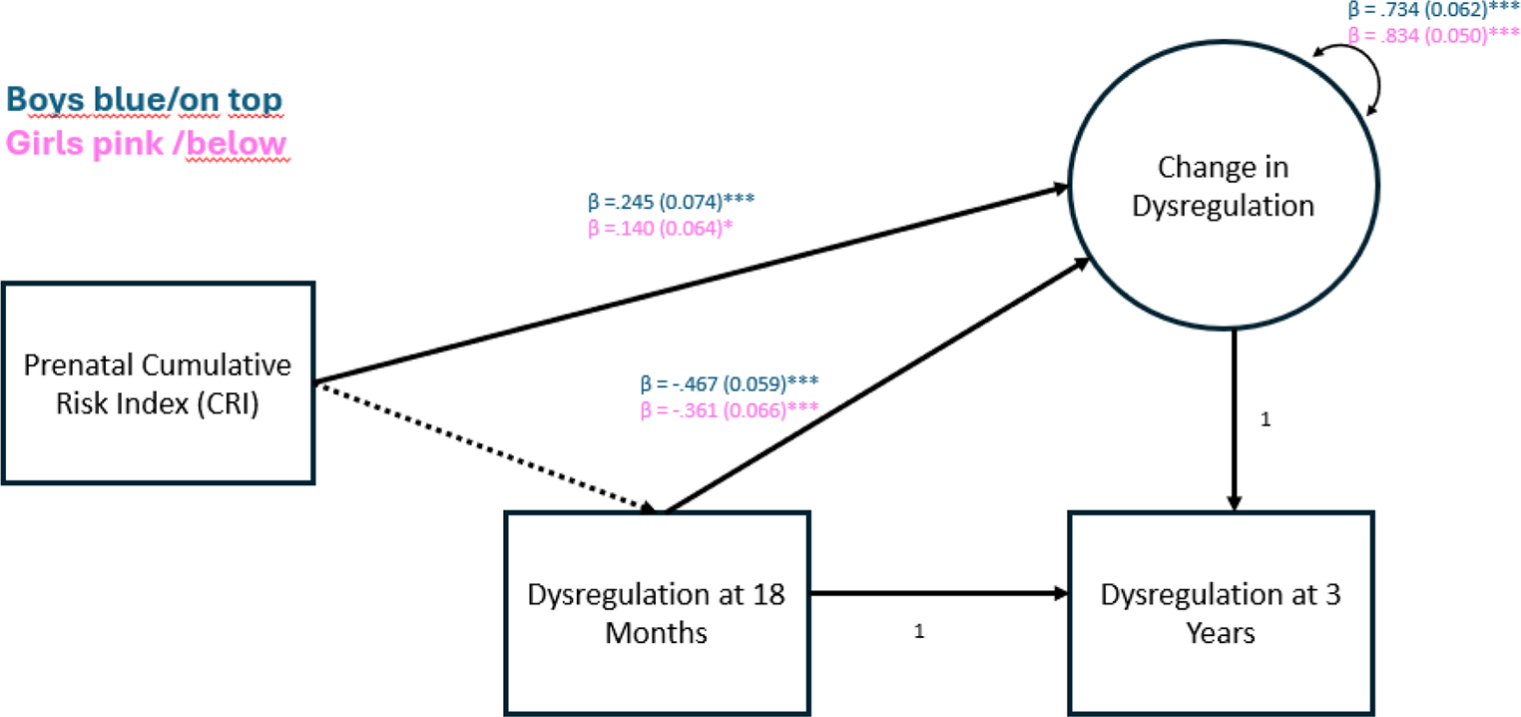
Prenatal cumulative risk and change in dysregulation from 18 months to 3 years for boys and girls. Note. Fitted model with parameters estimates, standard errors in parentheses, and variances for the change score

As to the second research question on whether child sex moderated the association between prenatal risks and escalated dysregulation, we first conducted preliminary analyses using independent sample t-tests. These did not reveal significant sex differences in terms of reported *levels* of dysregulation, neither at 18 months (*t*(640) = 0.908, *p* =.364), nor at 3 years (*t*(438) = 1.661, *p* =.098). In the main analysis, multigroup comparisons were performed with child sex as a grouping variable. In the unconstrained model, all parameters were allowed to be freely estimated across child sex. This was used as the comparison model for the restricted model in which parameters were constrained to equality across child sex. We found that paths from dysregulation at 18 months to the latent change factor were significantly different between boys and girls (Satorra Bentler χ2(1) = 5,07, *p* <.05), the variance of the change factor was also significantly different between sexes (Satorra Bentler χ2(1) = 8,05, *p* <.001). Finally, we found that the path from CRI to the change factor, controlling for gestational age, was significantly different for boys and girls (Satorra Bentler χ2(2) = 9,90, *p* <.01). Results for boys and girls respectively can be found in Fig. 1. As depicted, prenatal risk exposure affected boys` (*n* = 379) dysregulation development more negatively (*β* = 0.229, *p* =.003) than that of girls (*n* = 348) (β = 0.151, *p* =.017).

It should be noted that the effect sizes reported in this study were relatively small. Based on previous studies, this should be expected.

## Discussion

Higher cumulative risk during pregnancy predicted a greater increase in dysregulation from early toddlerhood (18 months) into the preschool years (3 years). This association was more pronounced for boys than for girls. Associations with gestational week or maternal age were not significant.

### Increases in Dysregulation Problems during Early Childhood Are Predicted by Prenatal Risks

Adressing the first research question on evolvement of dysregulation from 18 months to 3 years, exposure to elevated levels of risk in pregnancy was associated with an increase in regulatory problems between 18 months and 3 years of age. Hence, accumulated prenatal risks predicted increasing regulatory problems from early toddlerhood into the preschool years. In other words, our results suggest that the predictive power of prenatal cumulative risk on regulatory problems became increasingly more evident with time.

These results show that prenatal risk status is associated with a developmental trajectory of early dysregulation developing into increasing regulatory problems, while also supporting the notion that early dysregulation predicts later dysregulation (Fish et al., 1991; Olsen et al., 2019; Schmid et al., 2011; Sirvinskiene et al., 2012). As it is the persisting problems that elevate the child`s risk for long-term negative outcomes, being able to identify children and families at risk early is essential.

### Prenatal Risk Is Associated with Postnatal Risk

As Nelson and Gabard-Durnam (2020) point out, early adversity is often not time-limited. What constitutes effects of specific prenatal risk factors, interactive effects, or what can be considered sequala of cumulative risk over time, may be impossible to fully distinguish (Stein et al., 2014). Besides, *pre*natal risk is associated with *post*natal risk. For families that already have great levels of stress in their life before birth, having a baby with persistent regulatory problems may push the limits of existing coping resources, creating negative cascading spirals, and spill-over effects. Multiple maternal risks, in combination with a dysregulated toddler, could even accumulate additional risks postnatally. This entails that risk measured as early as the prenatal period may aid in identifying those at increased risk of developing persistent dysregulation problems in early childhood.

### Parental Behaviours, Capacity, and Depletion of Available Resources

Multiple explanations may be proposed for why the impact of prenatal factors became more pronounced from 18 months to 3 years of age. One possible explanation is through parenting capacity and parenting behaviours, and multiple studies point to parenting practices as mediators and moderators of cumulative risk on child outcome (Gach et al., 2018; Ruberry et al., 2018; Trentacosta et al., 2008). Failing to regulate the child could, for instance, foster a feeling of not being a good enough mother, affect parenting, interactional quality, and mental health negatively, creating negative transactional spirals due to an accumulation of distress over time. This may especially challenge at-risk mothers, who may have limited resources and support available to begin with (Sidor et al., 2017). Negative affect in the child in combination with poor or unsupportive regulatory strategies from the mother, may further hinder the child from developing internal regulation strategies of its own (Chan et al., 2021; Senehi & Brophy-Herb, 2020). It has also been suggested that infants and toddlers with multiple regulatory problems may be more susceptible to insensitive parenting, illustrating the hypothesis that dual risk in mother and child may reinforce each other negatively (Jaekel et al., 2021; Poehlmann et al., 2011). This also highlights the dyadic bidirectionality that saturates these early years, and that early child development in essence is relational.

As our study does not include postnatal measures of parenting, we cannot conclude in this regard. However, our results should be viewed considering this previous research, and parenting is one possible mechanism by which the reported associations could be understood.

### Prenatal Programming

Pregnancy and infancy represent a formative period for the developing nervous system (Porges & Furman, 2011), during which exposure to cumulative stressors may shape emerging dysregulation through neurobiological mechanisms such as HPA axis disruption and epigenetic modifications often termed “prenatal programming” (Nelson & Gabard-Durnam, 2020; Stein et al., 2014; Weinstock, 2008). Indeed, early childhood cumulative risk has been associated with decreased global brain measures and cortical thickness in children (Chad-Friedman et al., 2021). Long-term consequences for the child seem to depend on prenatal intensity and duration of exposure, genetic make-up, along with postnatal factors, such as maternal attention in interactions, and other environmental variables (Weinstock, 2008). Prenatal risk exposure could also exacerbate already latent vulnerabilities within the child (Bale & Epperson, 2015). As such, the children in our study that were exposed to a greater number of stressors during pregnancy, may also have had a disadvantageous neurobiological starting position for self-regulation. However, the absence of associations with dysregulation at 18 months in our study suggests limited support for early acting programming effects. Rather, the prediction of change from 18 months to 3 years may reflect a delayed or unfolding influence of prenatal risk, possibly interacting with postnatal environments.

### Sex Differences in Response To Prenatal Risk Exposure

The second research aim was to investigate whether child sex moderated the association between prenatal risks and escalation of dysregulation from toddlerhood into the preschool years. Previous research within this area is inconsistent. In terms of reported *levels* of dysregulation, boys and girls in our study did not differ significantly, neither at 18 months nor at 3 years. But results showed that cumulative risk during pregnancy predicted a greater increase in dysregulation from 18 months to 3 years for boys than for girls. Hence, prenatal risks accounted for more of the variance in pathways of increasing dysregulation for boys than it did for girls. For girls, other factors may play a greater role.

The sources of these sex differences cannot be identified by this study, and further investigations are needed. Some potential interpretations may include differential neurodevelopment and differences in genetic prenatal programming for girls and boys respectively (Bale, 2011). A second possibility is to consider gender-typed interaction patterns postnatally. For instance, some evidence suggests that boys rely on more external help regulating than girls (Tronick & Weinberg, 2000), and that the repairing of errors in interactions takes longer for boys (Weinberg et al., 1999), requiring prolonged regulation efforts from the parent. But if an environment characterized by multiple stressors provides less external support, this could play out differently for boys and girls over time. A third option is to consider how societal gender norms influence parental reporting. With traditional gender roles, aspects of dysregulated behaviour that may be considered within the normal range for boys, may be reported as problematic for girls. This could mean that boys` problems are comparatively more serious when reported by parents, and thus more likely to be associated with prenatal risk factors.

## Limitations

As is always the case with longitudinal research, attrition may be problematic, especially if the drop-out is selective. In this study attrition was to some extent related to CRI and maternal age. This may limit generalizability. However, by using FIML we hope to reduce such biases. The findings need replication on more diverse samples.

There are also several challenges associated with calculating and applying a cumulative risk index. The dichotomization and equal weighting of risk factors create obvious limitations (Evans et al., 2013). This approach offers no information on risk intensity, duration, nor nature of the risk beyond cut-off values (Ettekal et al., 2019), and essentially is a variable-centred approach. While other, more person-centred approaches could have provided insights into subgroups or constellations of risks, the cumulative risk approach was considered better suited to address the study aim. Although creating an index based on the combination of continuous and dichotomous indicators clearly is a complicated matter, it does however allow for aggregating various types of risks into a parsimonious model (Ettekal et al., 2019; Evans et al., 2013). As we aim to provide a holistic overview of overall risk status, dichotomizing the indicators is a way of addressing this. Naturally, a risk index does not include every possible risk factor that affects child or parental functioning. Moreover, the CRI is composed of risk factors only. Exposure to protective factors may be just as important in shaping child development (Rutter, 1979). Additionally, this approach is not suited to uncover underlying mechanisms, it does however allow us to identify families at elevated risk at a very early time point.

Thirdly, the lack of postnatal measures, including postnatal CRI scores, prevents us from understanding the underlying mechanisms at play. Interpretations are therefore limited to prediction.

Also, measures of dysregulation were based on maternal reports. Although this is the most frequently used way to measure such problems (Hemmi et al., 2011), characteristics of the mothers may colour their perception of their children (Najman et al., 2000) and cause bias in reports.

Finally, internal reliability for the sensory sensitivity subscale was on the lower side, and the interpretation of this particular construct should be interpreted with caution. Note, however, that reliability for the full dysregulation domain was good at both measurement points (0.76 and 0.83 respectively). Despite limitations, our measure of dysregulation (ITSEA) is a comprehensive and well-validated questionnaire, which, ultimately, represents a significant strength.

### Clinical Implications

Given the high prevalence of regulatory problems, as well as the strain such problems pose on child, parent, parent-child-relationship, and the family context as a whole, a better understanding of this phenomenon is of great clinical importance (Singh et al., 2021). As highlighted above, these problems present a well-known concern in clinical work with families. The findings underscore the importance of attending to *very* early risk factors in elucidating the developmental pathways leading to child dysregulation. Our results show that an accumulation of prenatal stressors is associated with the development of regulatory capabilities from early toddlerhood and into the preschool years, especially among boys.

Still, effect sizes are small, and it is likely that accumulated risk in pregnancy also interacts with postnatal factors in complex and significant ways. This places some restrictions on what clinical implications can be drawn. However, two main implications should be emphasized:

First, addressing dysregulation in young children may require a holistic approach, not solely focusing on the dysregulated behaviours of the child, nor on specific risk factors in the parent. This resonates well with previous work underlining the need to alleviate stress in both child and parent, as well as promoting parental self-regulation, and co-regulation skills (Singh et al., 2021). This is especially important as dysregulated children and parents are at risk for reinforcing each other negatively (Jaekel et al., 2021), and because multiple regulatory problems tend to compromise parent-child relationships, as well as relationships between parents (Singh et al., 2021).

Second, our study finds that regulatory issues may increase from toddlerhood into the preschool years, particularly among families facing multiple risks. Therefore, it is important to focus on dysregulation in this age group, and to provide long-term support when necessary. In Norway, frequent check-ups are offered the family during the baby`s first year of life and is then gradually reduced. For families of children with multiple regulatory problems, it might prove necessary to prolong the period of frequent follow-up. However, as these parents are often overwhelmed and exhausted, frequent appointments could also be challenging to attend. Consequently, seeking to reduce barriers to available help and providing flexibility in services, for example through home visits, may be key.

Future studies should investigate how prenatal cumulative risk interacts with postnatal factors and underlying mechanism of persistent dysregulation problems in early childhood. It is important to determine if dysregulation stems from system overload, which can be managed by lowering risk and boosting protective factors, or if it requires specific interventions. A better understanding of underlying mechanisms will enable more targeted interventions and clinically actionable approaches.

## Electronic Supplementary Material

Below is the link to the electronic supplementary material.

Supplementary Material 1

## Data Availability

Data can be made available upon reasonable request.

## Footnotes

1 Snus is a non-combustible nicotine product frequently used in the Nordic countries.
