# Supplementary material for: Mapping Dysregulation: Prenatal Predictors and Developmental Trajectories of Multiple Regulatory Problems in Early Childhood
Source: Res Child Adolesc Psychopathol. 2025 Apr 8;53(8):1155–68. doi: 10.1007/s10802-025-01320-2 (PMC12357804; doi:10.1007/s10802-025-01320-2)

Figure S1. Unconditional Latent Change Score Model for Prenatal Cumulative Risk Index in Relation to Dysregulation at 18 Months and 3 years, *n* = 748.


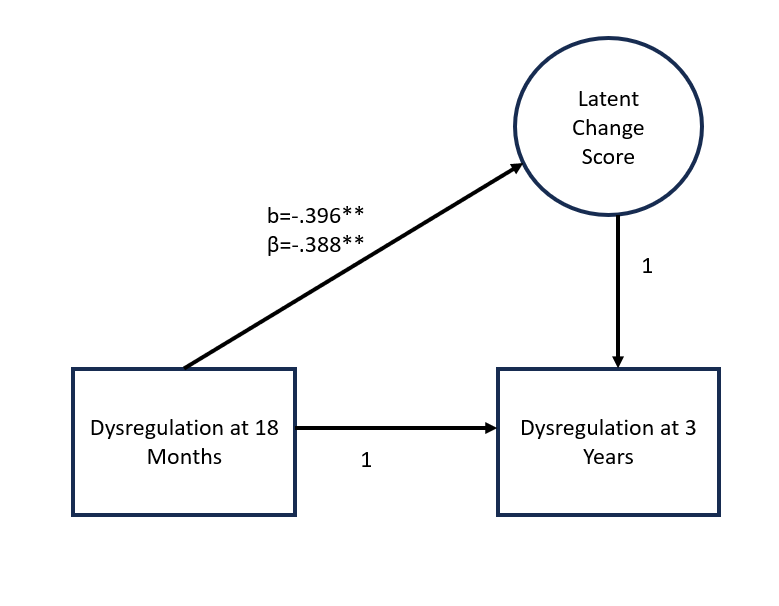

Supplement: Supplementary file 1 — Supplementary Material 1 [file 10802_2025_1320_MOESM1_ESM.docx]
